# Haemopoietic stem cell transplantation in Systemic lupus erythematosus: a systematic review

**DOI:** 10.1186/s13223-019-0373-y

**Published:** 2019-09-18

**Authors:** Nipun Lakshitha de Silva, Suranjith L. Seneviratne

**Affiliations:** 1 Department of Clinical Sciences, Faculty of Medicine, General Sir John Kotelawala Defence University, Colombo, Sri Lanka; 2 Department of Surgery, Faculty of Medicine, University of Colombo, Colombo, Sri Lanka; 3 Institute of Immunity and Transplantation, University College London and Royal Free Hospital, London, UK

**Keywords:** Systemic lupus erythematosus, Bone marrow transplant, Haemopoietic stem cell transplant, Morbidity, Mortality

## Abstract

Despite advances in treating Systemic lupus erythematosus (SLE), a proportion of patients continue to face significant morbidity and mortality. Haemopoietic stem cell transplant (HSCT) has been recognized as an option for such patients. We analysed the evidence on efficacy and safety of HSCT in patients with SLE. A database search was done for articles on HSCT in SLE up to July 2017 in PUBMED, Cochrane library, LILACS and clinical trial registration databases to select prospective or retrospective studies with 8 or more patients. Of the 732 search results from the PUBMED, Cochrane and LILACS database search, following duplicate removal, 15 studies were eligible for detailed assessment. Findings of an additional trial were obtained from the clinical trial registration database. Data were extracted on study design, patient characteristics, nature of intervention, outcomes, complications and study quality. Case reports and small case series were summarised without detailed qualitative analysis. Most of the studies showed remission in the majority of patients. Relapse of the original disease increased with longer follow-up. Common adverse effects included: infections and secondary autoimmune disorders. Short follow up period and lack of randomised controlled trials were the main limitations restricting the generalizability of study results. A meta-analysis was not performed due to heterogeneity of studies. Although HSCT is a viable option in SLE, its exact clinical utility needs to be further evaluated in well-designed studies.

## Background

Systemic lupus erythematosus (SLE) is a multi-system autoimmune disorder which commonly runs a lifelong clinical course. It has a complex pathogenic process, heterogenous clinical presentations and a varying range of severity from mild disease to severe life threatening multi-organ involvement. SLE is seen worldwide though its prevalence is higher among Afro-Carribean and South Asian individuals [1]. Disease prevalence ranges from 20 to 150 per 100,000, and both the incidence and prevalence has been rising over the past decades [1]. This is partly due to improved diagnostics and survival due to better treatment modalities. It is very likely that clinicians would encounter more SLE patients needing advanced treatment strategies in the future. SLE produces significant morbidity and mortality. This may be related to organ involvement (skin, joint, kidney, blood, nervous system, mucosal surfaces) in the disease, as well as treatment complications and co-morbidities such as cardiovascular disease or osteoporosis. Mortality is around 15/1000 person years, which is more than 60% higher than in controls [2].

Treatment of SLE is complex and needs a multidisciplinary approach. Pharmacological treatment in the form of immune suppression (steroids and steroid sparing agents such as azathioprine, mycophenolate mofetil and calcineurin inhibitors) plays an important first line role [3]. In addition, Hydroxychloroquine has many beneficial effects. In patients showing a poor response to above drugs or experiencing major side effects, biologics are being increasingly used. Despite all these therapeutic measures, a significant proportion of patients continue to have high disease activity and relapse frequently with resultant organ damage. The inherent heterogeneity in disease mechanisms has made it difficult to design therapies that work effectively in most if not all SLE patients [3]. Thus, new management options targeting patients with severe or refractory disease are needed. Haemopoietic stem cell transplant (HSCT) has been tried in patients with SLE over the last two decades. Though its use is rising in this patient group, it has still not been recognized as a standard treatment option. We systematically analysed the available evidence from prospective or retrospective studies and large case series with the objective of assessing efficacy and safety of HSCT in SLE patients, so as to define if this may be considered as a potential therapeutic option in clinical practice.

## Main text

### Methods

We designed the study to review prospective or retrospective studies on HSCT in patients with SLE for any indication. We searched PUBMED, Cochrane library and LILACS databases for indexed publications. We searched PUBMED with no restriction in time or type of article for ‘SLE’ OR ‘lupus’ with any of the terms ‘stem cell transplantation’ OR ‘bone marrow transplantation’ in all fields with language restriction to English. Last date of search was 31^st^ July 2017. A similar strategy was used for other databases. Subsequently, the search was expanded to clinical trial registries and databases including International Standard Randomised Controlled Trials Number (ISRCTN), International Clinical Trial Registry Platform (ICTRP) and clinicalTrials.gov of National Library of Medicine using similar search strategies. References provided in full text articles, were also used to identify additional references and articles. Case reports and case series with less than 8 patients were excluded prior to detailed analysis to minimise overrepresentation of individual cases.

Both authors independently reviewed the abstracts and selected the studies to review the full articles. All full articles were reviewed by the two authors independently and entered a summary into a pre-defined data base. Study design, patient characteristics, nature of intervention, outcomes, complication and study quality were assessed by the investigators. Primary outcome measures of clinical importance included clinical remission, relapse and clinical disease activity scores. Mortality was assessed as a secondary outcome. Study quality was determined based on study design, sample size, presence of pre-determined patient inclusion criteria and outcome measures and period of post-transplant follow up. Risk of bias of individual studies was planned to be analysed in case of controlled studies. The final database was developed by the two investigators by consensus. A meta-analysis or assessment of risk of bias across studies were not performed in the presence of heterogeneity of the studies and in the absence of appropriate objective outcome measure across all the studies.

### Results

**Fig. 1:**
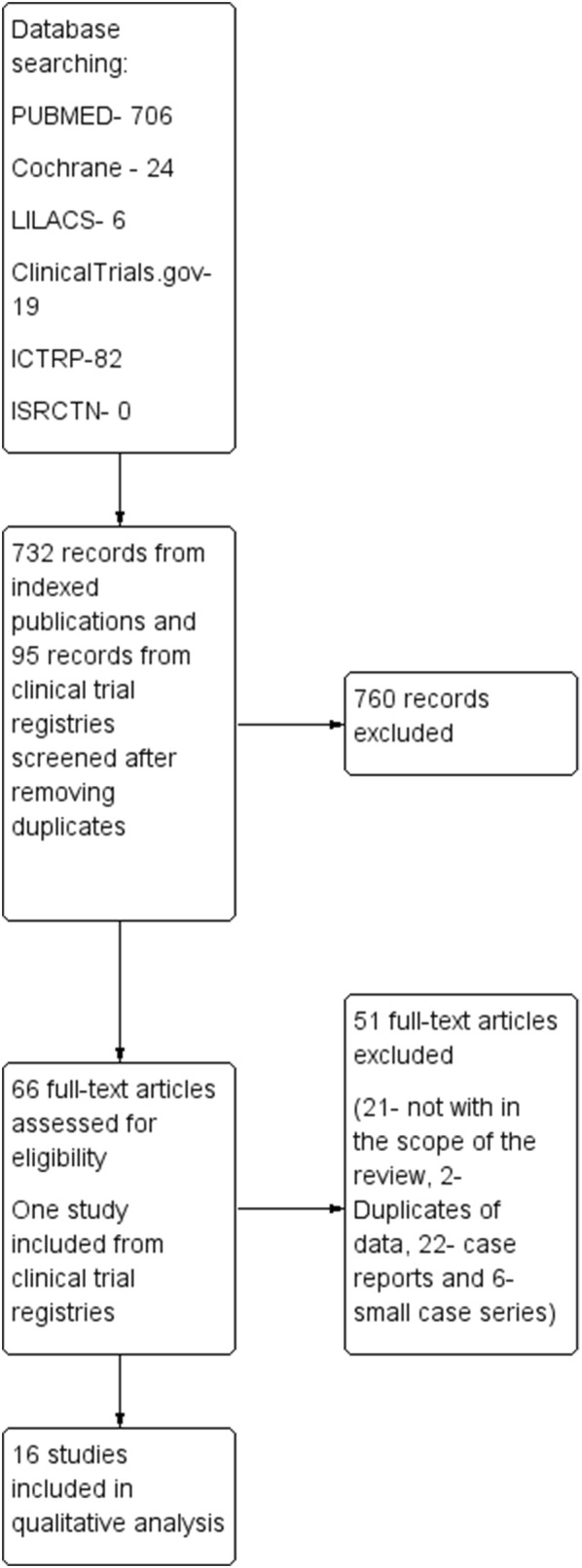
PRISMA diagram. PRISMA diagram of the study shows the methodology of selecting studies for the review. Results of searches from indexed databases (PUBMED, LILACS and Cochrane) and clinical trial registries (ClinicalTrials.gov, International Standard Randomised Controlled Trials Number -ISRCTN, International Clinical Trial Registry Platform—ICTRP) are shown parallel. After screening 732 entries from indexed databases and 95 entries from clinical trial registries 16 studies were included in qualitative analysis. Further 22 case reports and six small case series summarised

An initial search yielded 706 hits from ‘PUBMED’. Search in LILACS database yielded only six articles and Cochrane database search yielded 24 papers. Following removal of duplicates, 732 abstracts were screened for eligibility. After reviewing the abstracts, 66 papers were selected for further evaluation. One paper was removed due to duplication of data. Twenty-one larger studies/case series (with three or more patients) and twenty-two case reports (with one or two patients) were identified as relevant for the review. After excluding case reports and smaller studies with less than 8 patients (n = 6), 15 studies were selected for further detailed analysis. There were no randomized controlled studies. In the absence of any published randomised clinical trials, we expanded the search to clinical trial registries. ClinicalTrials.gov database and ICTRP searches yielded 19 and 82 hits respectively while there were no studies in ISRCTN database. Ninety-five trial registry entries were screened after removing the duplicates. Only one study that was not found in our initial search strategy was identified. The process of study selection is summarised in Fig. 1.

Of the 22 studies that were considered [4–25] only 16 studies with eight or more patients were analysed in detail and summarised in Table 1. This included 380 SLE patients with two studies having control group totalling a number of 59. Outcome was expressed in several ways, with maintenance of remission and SLE disease activity index (SLEDAI) being commonly used. Details of clinical outcome including efficacy and safety could not be aggregated since the patient characteristics, procedure, follow up duration, measures of outcome including definition of remission and relapse and use of immunesuppression varied markedly between studies. Important aspects of individual studies are outlined below.

**Table 1:** Summary of larger studies on Haemopoietic stem cell transplantation in Systemic lupus erythematosus

Study (reference)	Year	Country	Study design	Patient characteristics	Intervention^a,b,c^	Outcome	Complications
Traynor [5]	2000	USA	Case series	Nine patients with SLE with life or organ threatening complications not responding to several cycles of CYC (SLEDAI 17 to 37)	Autologous. MPP added to the conditioning regime	Seven patients remained in remission. No flares during follow up for > 1 year. SLEDAI score 0–5. No patient was on any immunosuppression other than a small dose of prednisolone	Two patients developed infection after stem cell harvest and one died. They did not undergo transplantation and were not included the in analysis. Two patients developed dermatomal herpes zoster and one developed pneumocystis pneumonia that resolved with treatment
Traynor [6]	2002	USA	Case series	Fifteen patients with SLE with organ impairment who failed to achieve sustained remission with monthly pulsed CYC and steroids	Autologous. MPP added to the conditioning regime	SLEDAI score decreased to 5 or < 5 in 12 patients. Twelve patients were followed up for > 1 year and immunosppressants were discontinued in 10 patients. Two patients relapsed with longer follow up (> 30 months)	Neutropenic fever without culture positivity. No mortality. Two patients underwent harvesting but died before conditioning; one due to fungal infection, other due to lupus cerebritis
Voltareli [7]	2002	China	Preliminary reporting	Nanjing group: ten Zhengzhou group: eighteen patients Beijing group: eight Shandong group: one (no further details)	All patients autologous. Nanjing: first three-conditioning with cytoxan and melphalan, used bone marrow. Next seven-CYC and ATG, Zhengzhou: mobilisation with CYC and ATG, bone marrow in six. No manipulation of stem cells. For conditioning total lymphoid irradiation added. Beijing group: Conditioning with CYC and TBI in four. Shandong: No details	Nanjing: eight remission, one stabilised, Zhengzhou: Complete remission in 12, partial remission in three and no remission in three. Beijing: all developed remission, Shandong: patient relapsed	Nanjing: one death due to pulmonary hypertension and cardiac failure. Beijing: Three developed CMV infections
Jayne [8]	2004	Europe	Retrospective registry survey	Fifty-three patients with SLE	Autologous. In three mobilisation only with CYC or G-CSF. Source of stem cells peripheral (77%) or bone marrow (21%) or both (2%). No graft manipulation in 28. Conditioning different regimes	Out of 50 evaluable patients at 6 months 33 (66%) developed remission with SLEDAI < 3, seven (14%) developed partial remission. Ten patients developed relapse	Thirty-one severe or life threatening adverse events occurred in 28 patients including 22 infections (three deaths), five immune events, two malignancies (one death), four deaths due to progressive disease and seven deaths related to procedure
Statkute [10]	2005	USA	Retrospective analysis	Twenty-eight patients with SLE with organ involvement and evidence of APLS. (Out of 46 patients who underwent HSCT)	Autologous	Twenty-one patient entered remission and nine were able to maintain remission after discontinuing all medicines. Twenty-two were on anticoagulation. After discontinuing anticoagulation in 18, 14 were free from thrombosis	No treatment related mortality. Infection during hospital stay in nine patients
Burt [11]	2006	USA	Single arm trial	Fifty patients with organ or life-threatening visceral involvement due to SLE requiring minimum 20 mg/day prednisolone or equivalent while on CYC	Autologous	Disease free survival at 5 years 50%. Overall survival at 5 years 84%. Four patients never entered remission. Majority showed improvement of disease activity	Two deaths prior to transplantation (One due to mucormycosis, other due to active disease). Six deaths after transplant not related to transplant (Four due to active SLE). Non-fatal infections were common during stem cell mobilisation and transplantation period
Loh [12]	2007	USA	Retrospective	Out of 55 patients with SLE thirteen patients with cardiac involvement (LV dysfuction—6, pulmonary hypertension—5, mitral valve disease—3, large pericardial effusion—1)	Autologous. CD 34+ cells selected/unmanipulated stem cells. Conditioning CYC with ATG or alemtuzumab	Patients with mitral valve disease and pericardial effusion improved. All patients with LV dysfunction had at least minimal improvement. Only two patients with pulmonary hypertension had stable or improved pulmonary pressure	Three patients (two with LV dysfunction, one with pulmonary hypertension) died
Vanikar [13]	2007	India	Retrospective	Twenty-seven patients with lupus nephritis	Allogenic from related donors. Unfractionated HSC transplanted into peripheral circulation, thymus and bone marrow. Non myeloablative low intensity conditioning done.	Average follow up 4.9 years. Average disease free interval 7.35 months	No GVHD. No life threatening side effects. Two deaths more than 2 years later
Gualandi [14]	2007	Italy	Case series	Eight patients with SLE (further information not available)	Autologous. Conditioning with CYC and thiotepa	All patients achieved complete remission. Two patients relapsed, but well controlled with treatment. Cumulative SLEDAI from 90 to 9	No significant adverse events
Meng [17]	2011	China	Controlled, non-randomized	HSCT-11, SLEDAI 20 ± 5, non-transplant—39, SLEDAI 21 ± 3	Autologous HSCT Vs. immunesupression and assessed outcome in pregnancy after remission	HSCT-SLEDAI preconception 4 ± 1, postpartum 4 ± 1, no lupus nephritis/hypertension during pregnancy, non transplant-SLEDAI preconception 4 ± 2, postpartum 7 ± 2, 33% hypertension and 31% lupus nephritis during pregnancy	Side effects related to treatment not described
Song [18]	2011	China	Controlled, non-randomised	Intervention—17 patients, Control—20 patients	Intervention-autologous HSCT, Control-conventional therapy (Steroids and CYC, three patients only steroids, four patients MMF added)	Intervention—16/17 steroids stopped within one year. SLEDAI in 5 years 32.3 ± 9.2 to 0.76 ± 0.92 (p < 0.01), Control—15/20 achieved remission, SLEDAI in 5 years 18.21 ± 5.71 to 6.28 ± 4.48 (p < 0.01)	Intervention—two patients died at 33 and 64 months due to severe pneumonia and heart failure, Control—Five patients died 2–74 months, nine had disease flares
Pasquini [19]	2012	North and South America	Retrospective	SLE	Autologous—27 patients, Allogenic—3 patients	Median follow up 31 months for autologous group. No adequate data on disease outcome	Autologous—8 died (6—infection, 1—disease relapse, 1—graft failure), Allogenic—1 died due to infection
Alchi [21]	2012	Europe	Retrospective	Twenty-eight patients with SLE	Autologous. Unmanipulated stem cells in 18. Conditioning regime low [10], moderate [18] intensity	Median follow up 38 months. Five year overall survival 81 ± 8%, relapse incidence 56 ± 11%, disease free survival 29 ± 9%	Thirty-one severe or life threatening adverse events. Two secondary autoimmune disorders and one lymphoproliferative disorder. Five deaths within 2 years after transplant, three due to infections, one due to secondary autoimmune disorder and one due to progressive SLE
Leng [23]	2017	China	Non-controlled, non-randomized	Severe SLE—27 patients, 3 removed from analysis due to inadequate stem cell harvesting (n = 1) and being lost to follow up (n = 2)	Autologous. Conditioning CYC with ATG/ALG/TBI	6 months—two partial remission, 21 remission, 10 years-one remained active, four lost to follow up, 16 remained in remission, 14 with lupus nephritis 4 g/24 h pre-treatment to 0 g/24 h at 5 and 10 years	Three patients died (23 days, 19 months, 3 years), 8 patients developed CMV infection
Cao [24]	2017	China	Prospective non-controlled	Twenty-two patients with lupus nephritis and failed previous therapy or other significant organ involvement	Autologous	10 patients relapsed within 10 years of median follow up. Five year progression free survival was 67.9%	One patient died due to infection several years post-HSCT. Several patients developed opportunistic infections and 13 had CMV reactivation. Three developed secondary autoimmune disease and two had malignancies
Clinical trials.gov [25]	2017	USA	Prospective non-controlled	Eight patients with severe, active lupus refractory to immunesuppression between 15 and 40 years	Priming with rituximab, MPP and CYC. Conditioning with fludarabine, rituximab and CYC	In follow-up up to 3 years two patients have maintained complete remission with SLEDAI 0	Six out of eight patients died within 1 year

This table summarises prospective and retrospective studies and larger case series on Haemopoietic stem cell transplantation in Systemic lupus erythematosus. Country of the study, study design, participant characteristics, intervention, outcome and complications are outlined under each study

*ALG* anti-lymphocyte globulin, *APLS* anti phospholipid syndrome, *ATG* anti-thymocyte globulin, *CYC* cyclophosphamide, *G-CSF* granulocyte colony stimulating factor, *MPP* methylprednisolone, *SIRS* systemic inflammatory response syndrome, *SLEDAI* SLE disease activity index, *TBI* total body irradiation

^a^Drugs used for mobilisation not mentioned if CYC and G-CSF was used. Other regimes specified

^b^Drugs used for induction not mentioned when CYC and ATG. Other regimes specified

^c^Cell selection if done other than through leukapheresis from peripheral circulation and subsequent CD 34+ separation it was specified

In 2000, Traynor et al. described their experience with nine SLE patients having life threatening disease who were recruited for autologous HSCT. Two of them were excluded post-harvesting due to infection though it is not clear whether they received immunesuppression [5]. The first patient described in this case series had similar characteristics to the patient described in more detail in a separate case report published in 1999 [26]. Though this is a case series with only nine patients it had included severe patients with multiple organ involvement with very severe refractory disease and showed objective clinical improvement with reduction of SLEDAI score to < 5. Three were not on any immunosuppressive while three were tapered to have prednisolone 5 mg/day. Study methodology was also described well though longer follow up would have added to the quality of study.

The next study in 2002 is a case series of 15 patients with refractory SLE [6]. There were two more patients who underwent harvesting, but excluded since they died prior to completion of HSCT without receiving immunoablative therapy. This study was well designed with clear patient selection criteria, uniform methodology across all patients and clear outcome measures. However the follow up has varied from 2 to 66 months. While all 15 patients achieved remission two have relapsed with longer follow up. Seven patients were on steroids, up to prednisolone 30 mg/day and one patient was on cyclophosphamide. Others were not on any immunosuppressive.

In a preliminary report of HSCT for autoimmune diseases in China and Brazil, information on the group of patients from Brazil is inadequate for assessment of outcome though four patients had SLE [7]. Available information from centres from China shows a favourable response though detailed assessment of long term follow-up and complications cannot be made. Patient characteristics are not adequately available and transplant methods vary significantly between patients and centers. There is no information on concomitant immunosuppression or disease activity scores.

Retrospective data collected from 23 centres reporting to EBMT/EULAR registry reports 53 patients who received autologous HSCT for SLE [8]. This series includes a heterogeneous patient population with different methodologies used in the HSCT. While a significant proportion achieved remission, mortality rate remains high in relation to procedure (12%, 95% CI 3–21%) with 10 patients developing relapse and 70% of the patients continuing immunosuppression. The mortality rate was associated with long duration of disease activity prior to the HSCT. Though this describes a large sample size its study design, methodological features and markedly variable follow up period from 0 to 78 months reduce the validity of results.

A retrospective review from USA describes 28 patients with Antiphospholipid syndrome (APLS) out of a larger cohort of 46 SLE patients who have undergone HSCT [10]. Though this describes a selected group of patients with potential for reporting bias patient characteristics, methodology and outcomes are very clearly defined. Follow up varied between 6 and 78 months. Of the 21 patients who achieved remission only 10 were on immunosuppression which was 10 mg or less prednisolone daily. Majority were able to discontinue anticoagulation without recurrent thrombosis.

Burt et al. [11] reports a single arm trial which was well-designed and included 50 patients with SLE and life threatening visceral involvement showing good remission rates during follow up varying from 6 months to 7.5 years. However there were 8 deaths, mostly related to severe disease activity. Use of concomitant immunosuppression is not clearly described though a prednisolone dose > 10 mg/day was considered as inability to achieve remission. Disease free survival was only 50%. In a retrospective report of 13 patients with SLE and cardiac involvement who have undergone HSCT authors describe the cardiac outcome of the patients [12]. All patients had other system involvement due to SLE as well. Two died due to SLE progression/relapse and one due to an accident, seven had improvement in SLE and cardiac condition during follow up ranging from 8 to 105 months. Retrospective nature and small number with heterogenous patient population minimise ability to make conclusions based on the results, however this at least shows the safety of the procedure in patient with cardiac involvement and the potential for resolution in cardiac involvement.

A retrospective study from India covering patients with Pemphigus vulgaris and SLE reports data from 27 patients with lupus nephritis who underwent allogenic HSCT [13]. Baseline clinical information of the patients is inadequate and authors report a disease free interval of 7.35 months with no clear data on remission and relapse. Two deaths occurred more than 2 years later and the cause is not mentioned. A case series from Italy including 8 patients with SLE who have undergone HSCT shows remission in all though two relapsed subsequently [14]. Patients’ clinical details are minimum and the procedure variations among patients are seen. Follow up periods of individual patients are not available separately.

Pregnancy in SLE patients was particularly studied by a group of investigators where pregnancy outcome was compared in patients who were managed with immunosuppression Vs. HSCT [17]. Though the preconception SLEDAI was comparable in two groups pregnancy outcome was superior in transplant group. However the method of enrolment into study, allocation to groups and timing of pregnancy with regards to HSCT of patients are not clear in methodology and there is potential for selection bias since well controlled SLE patients are only considered for pregnancy. Also if there is loss of fertility due to high dose cyclophosphamide in HSCT group that is masked in this study as people who have successfully conceived only could be included in this study.

In a controlled study comparing autologous HSCT vs. conventional therapy investigators have reported better outcome and reduced mortality with HSCT. [18]. However methodological limitations including lack of randomization and lack of information regarding matching of control patients were noticed. Also pre-transplant SLEDAI scores between two groups were not comparable. Despite the fact that patients with severe disease were in intervention group they had a better outcome. All could discontinue steroids except one who was on prednisolone 5 mg/day. Follow up period ranged from 33 to 110 months with a median of 89 months which is considerably long.

In a retrospective reporting on 368 patients who underwent HSCT for autoimmune diseases in North and South America there is information on 30 patients with SLE [19]. Since this data covers two patients reported in another paper that has not been again reviewed in this review [27]. This report provides information on mortality with a relatively high mortality rate, but patient characteristics, concomitant immunesuppression and clinical outcome are not described in detail. Another retrospective review describes 28 patients who were followed up for one to 110 (median 38) months [21]. This covers a heterogeneous group of patients and conditioning regime varied between patients. Most of the patients continued to be on immunesuppression post-transplant. With a disease free survival of < 30% at 5 years and high mortality this contrasts with some other studies showing much positive results.

Two recent publications from China report a series of patients who underwent HSCT for SLE and followed up for a long time up to 10 years [23, 24]. First study describes 24 out of 27 patients who had well defined inclusion criteria and followed up for median 10 years (14 followed up for more than 10 years). This provided satisfactory long term outcome, though post-transplant immunesupression continues to be significant and the dose range of steroids was not specified. Second prospective study describes 22 patients with lupus nephritis. Well defined inclusion criteria, long follow up (51–147 months, median 113 months) and detailed analysis of clinical outcome are strengths of this study. This study also provides evidence for excellent renal and overall outcome as well as low mortality though there were multiple non-fatal complications.

A clinical trial accessed through ClinicalTrials.gov (and not found as a full text article when searched in other databases) fulfilled eligibility criteria [25]. This study had enrolled 9 patients with severe SLE and major organ involvement. However, the last patient did not undergo transplantation and thus not included in the outcome analysis. There are differences in the methods of priming and conditioning compared to most of the other studies. The striking finding was an all-cause mortality of 6 patients, though further details for individual cases were not given.

In a retrospective study on secondary autoimmune diseases after HSCT for primary autoimmune diseases from European Blood and Marrow Transplantation (EBMT), authors reveal that out of 20 patients with SLE who underwent autologous HSCT five patients have developed secondary autoimmune diseases [28]. These study findings revealed that SLE being the primary autoimmune disease is a risk factor for secondary autoimmune diseases. There is scant data about patients who underwent allogenic HSCT. Other than occurrence of secondary autoimmune diseases other clinical outcomes were not described in above study. Therefore further in depth assessment was not performed.

Six small studies that were excluded from detailed analysis included a total of 27 patients with SLE with multiple different disease manifestations [4, 9, 15, 16, 20, 22]. One study included three patients with soft tissue calcification related to disease and multiple other complications who had excellent response to HSCT. Overall 18 patients sustained remission with eight having follow up for more than 5 years. Two other patients relapsed and seven other patients died (six due to immunosuppression related complications and one due to relapsed disease).

In 22 case reports, 24 patients are described [29–50]. Few of the initially reported patients have undergone HSCT for another condition co-existing with SLE [29, 32, 33]. As a result they were not enrolled with the intention of treating SLE. Seventeen patients maintained remission and four patients relapsed. Out of the patients who maintained remission one had 15 year follow up, another 5 year follow up and 11 patients had 1 to 5 year follow up. Others had a shorter follow up data. Three patients died and two deaths were related to severe sepsis whereas one patient died later related to complications of disease flare up. Other complications included sepsis, secondary autoimmune conditions, graft versus host disease and infusion reactions.

### Discussion

The available evidence suggests a trend towards benefit of autologous or allogenic HSCT in some SLE patients in achieving remission. This is specially so with regards to some recent well conducted studies with long term follow up data. Though there is scarcity of evidence regarding specific indications, conditioning regimes and concurrent medications from available literature, studies have overall enrolled patients with severe, organ or life-threatening disease while on high dose immunosuppression. Most important complications to anticipate include infections and secondary autoimmune diseases. Post-transplant deaths are mostly related to infections and relapse of the disease.

A significant proportion of patients either achieved complete or partial remission; however some relapsed within the study observation period. On the other hand, there was significant short term morbidity and mortality related to the procedure including opportunistic infections. Though most studies did not follow-up patients for a long duration, important long term effects observed in several studies include development of secondary autoimmune diseases and neoplasms including lymphoproliferative disorders.

The most important short term complication was opportunistic infections (CMV sepsis and fungal infections) with significant mortality. Intense immunosuppression as part of the conditioning regimes would increase the likelihood of opportunistic infections. The most important measures in preventing them would include standard nursing care of the immunosuppressed patient. Due to the heterogeneity of conditioning regimes and patient groups and the lack of randomised controlled studies, we were not able to define a specific conditioning regime that had a significantly lower risk of infections. Stringent measures to minimise opportunistic infections are crucial to ensure that benefit from any intervention outweighs the associated risks.

Development of secondary autoimmune diseases was a major long term complication of HSCT for SLE. These include autoimmune haemolytic anaemia, Evans syndrome, new onset APLS, acquired haemophilia and autoimmune thyroiditis. This had been difficult to manage in some of the cases. Since most of the studies have a short follow-up period when compared to studies in which the occurrence of secondary autoimmune diseases have been described, it is likely the incidence of this complication could be much higher than what is reported. A short interval between diagnosis and HSCT, ex vivo selection of CD 34+ cells and use of ATG have been suggested to be associated with a higher risk of secondary autoimmune disease [28]. Although the exact mechanisms of this complication are still poorly defined, the likely mechanisms includes imbalance between autoimmunity and tolerance during immune reconstitution following intense immunosuppression during conditioning as well as an inherent propensity of the patient to develop autoimmune disorders [51]. There might also be a loss of peripheral tolerance during conditioning [52], lympho-depletion by drugs used in conditioning and the proliferation of autoreactive T-cells [28]. Further studies with longer term follow up should provide a better understanding of these mechanisms.

Most of the studies included severe or refractory SLE patients who had already received treatment with potent immunosuppressants. At present, several biologics may also be tried as second line therapy in such patients. Rituximab has shown to be beneficial in patients with refractory SLE [53, 54]. The overall risk and cost of biologics would be less when compared to HSCT. As rituximab acts via B cell depletion it would not lead to pronounced immunoablation as during HSCT. There would still be a group of SLE patients who are refractory to all these agents.

It is postulated that HSCT induces remission in SLE patients through several mechanisms. High dose immunosuppression used during stem cell mobilization and conditioning would ablate several immune cells thus eliminating auto-reactive lymphocytes. Development and re-organisation of a self-tolerant immune system is also likely to contribute [24]. Studies on HSCT in SLE patients have found regulatory T cell numbers to return to levels seen in normal subjects. Furthermore, pathogenic T cell responses against auto-antigens are inhibited [55]. Whether these changes lead to adequate long term clinical remission and whether less intensive interventions may achieve such changes remain unanswered.

In four paediatric SLE patients resistant to conventional therapy, the efficacy of immunoablative high dose chemotherapy without HSCT was assessed. The authors found good outcomes of intensive chemotherapy alone [56] and made the argument that stem cells are resistant to the cytotoxic effects of high dose chemotherapy whereas lymphocytes are not. They postulated this therapy was immunoablative rather than being myeloablative, thus HSCT for haemopoietic reconstitution with the risk of reinfusion of autoreactive lymphocytes is not necessary. Furthermore, in a series of 14 patients with moderate to severe SLE resistant to conventional treatment, treating with high dose cyclophosphamide achieved complete response in five patients and partial response in seven patients [57].

There are few limitations that need to be considered in interpreting these studies. Risk of reporting bias with tendency to report cases with positive outcome, short follow up period of most of the studies and heterogeneity of patients and conditioning regimes are some of them. For example the study accessed via a clinical trial registry with no results published so far as a full text article showed high mortality [25]. Some studies excluded patients who were enrolled into HSCT and died or withdrawn following harvesting without undergoing HSCT leading to the risk of underestimating risks of procedure. Marked variations in outcome in different studies may be due to patient heterogeneity, the varying conditioning regimes used and the experience of the transplant centre.

A systematic review published in 2017 on autologous HSCT in SLE and APLS reviewed studies up to 2014 [58]. We have included studies up to 2017, including two large studies reported in 2017 that had the longest follow up. We have included several studies not included in the previous review (we have reviewed 44 studies in contrast to 25 studies in the previous publication). By doing so, we have been able to get a clearer idea about long term remission and complications such as secondary autoimmune diseases and lymphoproliferative disorders.

### Conclusions

Although there is a trend towards a positive risk benefit outcome for HSCT in SLE, strong evidence to support HSCT as a standard treatment strategy in SLE is still lacking. However, it would remain a viable option in selected SLE patients. Larger studies (ideally randomized and controlled) with longer follow up, comparing HSCT with high dose immunosuppression alone, biologics or conventional therapy should provide answers to many of the remaining questions.

## Data Availability

All data generated or analysed during this study are included in this published article

## References

[1] Lewis MJ, Jawad AS (2017). The effect of ethnicity and genetic ancestry on the epidemiology, clinical features and outcome of Systemic lupus erythematosus. Rheumatology (Oxford).

[2] Rees F, Doherty M, Grainge MJ, Lanyon P, Davenport G, Zhang W (2016). Mortality in Systemic lupus erythematosus in the United Kingdom 1999–2012. Rheumatology (Oxford).

[3] Davis LS, Reimold AM (2017). Research and therapeutics—traditional and emerging therapies in Systemic lupus erythematosus. Rheumatology.

[4] Rosen O, Thiel A, Massenkeil G, Hiepe F, Haupl T, Radtke H (2000). Autologous stem-cell transplantation in refractory autoimmune diseases after in vivo immunoablation and ex vivo depletion of mononuclear cells. Arthritis Res..

[5] Traynor AE, Schroeder J, Rosa RM, Cheng D, Stefka J, Mujais S (2000). Treatment of severe Systemic lupus erythematosus with high-dose chemotherapy and haemopoietic stem-cell transplantation: a phase I study. Lancet.

[6] Traynor AE, Barr WG, Rosa RM, Rodriguez J, Oyama Y, Baker S (2002). Hematopoietic stem cell transplantation for severe and refractory lupus Analysis after five years and fifteen patients. Arthritis Rheum.

[7] Voltarelli JC, Ouyang J (2003). Hematopoietic stem cell transplantation for autoimmune diseases in developing countries: current status and future prospectives. Bone Marrow Transplant.

[8] Jayne D, Passweg J, Marmont A, Farge D, Zhao X, Arnold R (2004). Autologous stem cell transplantation for Systemic lupus erythematosus. Lupus..

[9] Lisukov IA, Sizikova SA, Kulagin AD, Kruchkova IV, Gilevich AV, Konenkova LP (2004). High-dose immunosuppression with autologous stem cell transplantation in severe refractory Systemic lupus erythematosus. Lupus..

[10] Statkute L, Traynor A, Oyama Y, Yaung K, Verda L, Krosnjar N (2005). Antiphospholipid syndrome in patients with Systemic lupus erythematosus treated by autologous hematopoietic stem cell transplantation. Blood.

[11] Burt RK, Traynor A, Statkute L, Barr WG, Rosa R, Schroeder J (2006). Nonmyeloablative hematopoietic stem cell transplantation for Systemic lupus erythematosus. JAMA.

[12] Loh Y, Oyama Y, Statkute L, Traynor A, Satkus J, Quigley K (2007). Autologous hematopoietic stem cell transplantation in Systemic lupus erythematosus patients with cardiac dysfunction: feasibility and reversibility of ventricular and valvular dysfunction with transplant-induced remission. Bone Marrow Transplant.

[13] Vanikar AV, Modi PR, Patel RD, Kanodia KV, Shah VR, Trivedi VB (2007). Hematopoietic stem cell transplantation in autoimmune diseases: the Ahmedabad experience. Transplant Proc..

[14] Gualandi F, Bruno B, Van Lint MT, Luchetti S, Uccelli A, Capello E (2007). Autologous stem cell transplantation for severe autoimmune diseases: a 10-year experience. Ann N Y Acad Sci.

[15] Mandelbrot DA, Santos PW, Burt RK, Oyama Y, Block GA, Ahya SN (2008). Resolution of SLE-related soft-tissue calcification following haematopoietic stem cell transplantation. Nephrol Dial Transplant.

[16] Alexander T, Thiel A, Rosen O, Massenkeil G, Sattler A, Kohler S (2009). Depletion of autoreactive immunologic memory followed by autologous hematopoietic stem cell transplantation in patients with refractory SLE induces long-term remission through de novo generation of a juvenile and tolerant immune system. Blood.

[17] Meng J, Wang J, Liang W, Qin S, Wu C (2011). Long-term remission after successful pregnancy in autologous peripheral blood stem cell transplanted system lupus erythematosus patients. Rheumatol Int.

[18] Song XN, Lv HY, Sun LX, Meng JB, Wang JK, Zhang JQ (2011). Autologous stem cell transplantation for Systemic lupus erythematosus: report of efficacy and safety at 7 years of follow-up in 17 patients. Transplant Proc..

[19] Pasquini MC, Voltarelli J, Atkins HL, Hamerschlak N, Zhong X, Ahn KW (2012). Transplantation for autoimmune diseases in north and South America: a report of the Center for International Blood and Marrow Transplant Research. Biol Blood Marrow Transplant.

[20] Szodoray P, Varoczy L, Papp G, Barath S, Nakken B, Szegedi G (2012). Immunological reconstitution after autologous stem cell transplantation in patients with refractory systemic autoimmune diseases. Scand J Rheumatol.

[21] Alchi B, Jayne D, Labopin M, Demin A, Sergeevicheva V, Alexander T (2013). Autologous haematopoietic stem cell transplantation for Systemic lupus erythematosus: data from the European Group for Blood and Marrow Transplantation registry. Lupus..

[22] Su G, Luan Z, Wu F, Wang X, Tang X, Wu N (2013). Long-term follow-up of autologous stem cell transplantation for severe paediatric Systemic lupus erythematosus. Clin Rheumatol.

[23] Leng XM, Jiang Y, Zhou DB, Tian XP, Li TS, Wang SJ (2017). Good outcome of severe lupus patients with high-dose immunosuppressive therapy and autologous peripheral blood stem cell transplantation: a 10-year follow-up study. Clin Exp Rheumatol.

[24] Cao C, Wang M, Sun J, Peng X, Liu Q, Huang L (2017). Autologous peripheral blood haematopoietic stem cell transplantation for Systemic lupus erythematosus: the observation of long-term outcomes in a Chinese centre. Clin Exp Rheumatol.

[25] ClinicalTrials.gov [Internet]. Bethesda (MD): National Library of Medicine (US). 2000 Feb 29. Identifier: NCT00076752, Lymphocyte Depletion and Stem Cell Transplantation to Treat Severe Systemic Lupus Erythematosus; 2004. https://clinicaltrials.gov/ct2/show/results/NCT00076752. Accessed 31 May 2019.

[26] Traynor A, Burt RK (1999). Haematopoietic stem cell transplantation for active Systemic lupus erythematosus. Rheumatology (Oxford).

[27] Burt RK, Traynor AE, Pope R, Schroeder J, Cohen B, Karlin KH (1998). Treatment of autoimmune disease by intense immunosuppressive conditioning and autologous hematopoietic stem cell transplantation. Blood.

[28] Daikeler T, Labopin M, Di Gioia M, Abinun M, Alexander T, Miniati I (2011). Secondary autoimmune diseases occurring after HSCT for an autoimmune disease: a retrospective study of the EBMT Autoimmune Disease Working Party. Blood.

[29] Euler HH, Marmont AM, Bacigalupo A, Fastenrath S, Dreger P, Hoffknecht M (1996). Early recurrence or persistence of autoimmune diseases after unmanipulated autologous stem cell transplantation. Blood.

[30] Burt RK, Traynor A, Ramsey-Goldman R (1997). Hematopoietic stem-cell transplantation for Systemic lupus erythematosus. N Engl J Med.

[31] Marmont AM, van Lint MT, Gualandi F, Bacigalupo A (1997). Autologous marrow stem cell transplantation for severe Systemic lupus erythematosus of long duration. Lupus..

[32] Meloni G, Capria S, Vignetti M, Mandelli F, Modena V (1997). Blast crisis of chronic myelogenous leukemia in long-lasting Systemic lupus erythematosus: regression of both diseases after autologous bone marrow transplantation. Blood.

[33] Snowden JA, Patton WN, O’Donnell JL, Hannah EE, Hart DN (1997). Prolonged remission of longstanding Systemic lupus erythematosus after autologous bone marrow transplant for non-Hodgkin’s lymphoma. Bone Marrow Transplant.

[34] Musso M, Porretto F, Crescimanno A, Bondi F, Polizzi V, Scalone R (1998). Autologous peripheral blood stem and progenitor (CD34+) cell transplantation for Systemic lupus erythematosus complicated by Evans syndrome. Lupus..

[35] Fouillard L, Gorin NC, Laporte JP, Leon A, Brantus JF, Miossec P (1999). Control of severe Systemic lupus erythematosus after high-dose immunusuppressive therapy and transplantation of CD34+ purified autologous stem cells from peripheral blood. Lupus..

[36] Gur-Lavi M (1999). Long-term remission with allogenic bone marrow transplantation in Systemic lupus erythematosus. Arthritis Rheum.

[37] Trysberg E, Lindgren I, Tarkowski A (2000). Autologous stem cell transplantation in a case of treatment resistant central nervous system lupus. Ann Rheum Dis.

[38] Shaughnessy PJ, Ririe DW, Ornstein DL, Kissack B, Bickford DJ, Molina R (2001). Graft failure in a patient with Systemic lupus erythematosus (SLE) treated with high-dose immunosuppression and autologous stem cell rescue. Bone Marrow Transplant.

[39] Wulffraat NM, Sanders EA, Kamphuis SS, Rijkers GT, Kuis W, Lilien M (2001). Prolonged remission without treatment after autologous stem cell transplantation for refractory childhood Systemic lupus erythematosus. Arthritis Rheum.

[40] Brunner M, Greinix HT, Redlich K, Knobl P, Smolen J, Leitner G (2002). Autologous blood stem cell transplantation in refractory Systemic lupus erythematosus with severe pulmonary impairment: a case report. Arthritis Rheum.

[41] Besalduch J, Bargay J, Buades J, Galmes A, Morey M, Sampol A (2003). Autoimmune hemolityc anemia after treatment of severe Systemic lupus erythematosus with high-dose chemotherapy and autotransplantation of selected peripheral hematopoietic progenitors. Haematologica.

[42] Hashimoto N, Iwasaki T, Sekiguchi M, Takatsuka H, Okamoto T, Hashimoto T (2004). Autologous hematopoietic stem cell transplantation for refractory antiphospholipid syndrome causing myocardial necrosis. Bone Marrow Transplant.

[43] Talaulikar D, Tymms KE, Prosser I, Smith R (2005). Autologous peripheral blood stem cell transplantation with in vivo T-cell depletion for life threatening refractory Systemic lupus erythematosus. Lupus..

[44] Chen J, Wang Y, Kunkel G, Zhao H, Xue H, Xie X (2005). Use of CD34+ autologous stem cell transplantation in the treatment of children with refractory Systemic lupus erythematosus. Clin Rheumatol.

[45] Marmont AM, Gualandi F, van Lint MT, Guastoni C, Bacigalupo A (2006). Long term complete remission of severe nephrotic syndrome secondary to diffuse global (IV-G) lupus nephritis following autologous, haematopoietic peripheral stem (CD34+) cell transplantation. Lupus..

[46] Sweeney SE (2011). Hematopoietic stem cell transplant for Systemic lupus erythematosus: interferon regulatory factor 7 activation correlates with the IFN signature and recurrent disease. Lupus..

[47] Wada H, Terasako K, Kamiya Y, Sato M, Kimura SI, Okuda S (2011). Immune recovery after autologous PBSC transplantation without in vitro graft manipulation for refractory Systemic lupus erythematosus. Bone Marrow Transplant.

[48] Varoczy L, Kiss E, Tarr T, Zeher M, Szegedi G, Illes A (2012). Fatal CMV-Infection after Autologous Stem Cell Transplantation in Refractory Systemic Lupus Erythematosus. Case Rep Transplant..

[49] Alexander T, Schneider S, Hoyer B, Cheng Q, Thiel A, Ziemer S (2013). Development and resolution of secondary autoimmunity after autologous haematopoietic stem cell transplantation for Systemic lupus erythematosus: competition of plasma cells for survival niches?. Ann Rheum Dis.

[50] Gladstone DE, Petri M, Bolanos-Meade J, Dezern AE, Jones RJ, Fine D (2016). Long-term Systemic lupus erythematosus disease control after allogeneic bone marrow transplantation. Lupus..

[51] Loh Y, Oyama Y, Statkute L, Quigley K, Yaung K, Gonda E (2007). Development of a secondary autoimmune disorder after hematopoietic stem cell transplantation for autoimmune diseases: role of conditioning regimen used. Blood.

[52] Bohgaki T, Atsumi T, Koike T (2007). Multiple autoimmune diseases after autologous stem-cell transplantation. N Engl J Med.

[53] Iaccarino L, Bartoloni E, Carli L, Ceccarelli F, Conti F, De Vita S (2015). Efficacy and safety of off-label use of rituximab in refractory lupus: data from the Italian Multicentre Registry. Clin Exp Rheumatol.

[54] Serris A, Amoura Z, Canouï-Poitrine F, Terrier B, Hachulla E, Costedoat-Chalumeau N (2017). Efficacy and safety of rituximab for Systemic lupus erythematosus-associated immune cytopenias. A multicenter retrospective cohort study of 71 adults. Am J Hematol.

[55] Zhang L, Bertucci AM, Ramsey-Goldman R, Burt RK, Datta SK (2009). Regulatory T cell (Treg) subsets return in patients with refractory lupus following stem cell transplantation, and TGF-β-producing CD8+ Treg cells are associated with immunological remission of lupus. J Immunol..

[56] Luo XQ, Mo Y, Ke ZY, Xu L, Jiang XY, Zhang TT (2008). High-dose chemotherapy without stem cell transplantation for refractory childhood Systemic lupus erythematosus. Chemotherapy.

[57] Petri M, Jones RJ, Brodsky RA (2003). High-dose cyclophosphamide without stem cell transplantation in Systemic lupus erythematosus. Arthritis & Rheumatology..

[58] Leone A, Radin M, Almarzooqi AM, Al-Saleh J, Roccatello D, Sciascia S (2017). Autologous hematopoietic stem cell transplantation in Systemic Lupus Erythematosus and antiphospholipid syndrome: a systematic review. Autoimmun Rev.

